# Molecular docking analysis of HER-2 inhibitor from the ZINC database as anticancer agents

**DOI:** 10.6026/97320630016882

**Published:** 2020-11-30

**Authors:** Khalid Hussain Wali Sait, Mutaib Mashraqi, Asim Abdulaziz Khogeer, Othman Alzahrani, Nisreen M Anfinan, Hesham Khalid Sait, Abdulrahman Almutairi, Qamre Alam

**Affiliations:** 1 Department of Obstetrics and Gynecology, Gynecology Oncology Unite, Faculty of Medicine, King Abdulaziz University, Jeddah, Saudi Arabia; 2 Department of Clinical Laboratory Sciences, College of Applied Medical Sciences, Najran University, Najran 61441, Saudi Arabia; 3 Medical Molecular Genetics, General Directorate of Health Affairs Makkah Region, Ministry of Health (MOH), Saudi Arbia; 4 Department of Biology, Faculty of Science, University of Tabuk, Tabuk 71491, Saudi Arabia; 5 Genome and Biotechnology Unit, Faculty of Sciences, University of Tabuk, Tabuk 71491, Saudi Arabia; 8 Department of Pathology and Laboratory Medicine, King Abdulaziz Medical City,Ministry of National Guard Health Affairs (MNGHA), Riyadh, Saudi Arabia; 9 Medical Genomics Research Department, King Abdullah International Medical Research Center (KAIMRC), King Saud bin Abdulaziz University for Health Sciences, Ministry of National Guard Health Affairs (MNGHA), Riyadh, Saudi Arabia

**Keywords:** Human epidermal growth factor, Breast cancer, Drug development, Lapatinib

## Abstract

The human epidermal growth factor (HER2) is a transmembrane receptor that is highly expressed in breast cancer and in different other cancers. Therefore, it is of interest to identify the new HER2 inhibitors from a selected 300 compounds in the ZINC database. The top two hit compounds (ZINC000014780728 (-11.0 kcal/mol) and ZINC000014762512 (-10.8 kcal/mol)) showed a high affinity with HER2 relative to the reference compound (lapatinib (-10.2 kcal/mol)) for further consideration.

## Background

Breast cancer (BC) is one of the most prevalent causes of malignancies in women globally [1]. The incidences of BC in developed countries are comparatively higher relative to the underdeveloped countries [2]. Enhanced growth-promoting protein levels represent some BCs and are described as HER2 (Human Epidermal Growth Factor Receptor 2) cancers [3]. HER2 belongs to a superfamily of human peptide ligands epidermal growth factor receptors (EGFR) that consist of HER1, HER2, HER3, and HER4. EGFR and HER2 is the most promising therapeutic target for cancer [4]. HER2 is a 185-kDa protein, having an extracellular (ligand-binding) domain, a transmembrane domain, and a cytoplasmic tyrosine kinase domain. The ligand-binding domain (extracellular region) comprises of four domains (I-IV). Following the ligand binding, receptors get activated and lead to form the homo- and/or heterodimers of the receptors. The heterodimer of HER2 with HER3 is considered a highly potent oncogenic component due to the ligand stimulated tyrosine phosphorylation, and downstream signaling [5]. Besides, HER2 is the only exception that does not directly bind to any known ligands and undergo dimerization even in absence of a ligand [6].The control regulation of HER2 signaling is essential for normal cell growth and development [7]. The activation of the tyrosine kinase domain is a prevalent tumorigenesis mechanism [8] and has been linked with oncogenic activity in BC, and various other cancers [9]. Two major approaches for BC cure are the development of tyrosine kinase inhibitors and targeting the ligand-binding domains of HER2 by monoclonal antibodies that prevent the dimerization and subsequently the intracellular signaling cascade [10]. Lapatinib and gefitinib are the established inhibitor of HER2 that exert their activity by inhibiting the tyrosine kinase domain activation and have been approved for the management of BC and various other cancers [11,12]. Inhibition of EGFR family arrests the cell cycle progression and stimulates the apoptosis in different cancer model [13,14,15]. However, continued lapatinib administration may stimulate drug resistance, which warrants the development of new candidate drugs [16]. Therefore, it is of interest to identify the new HER2 inhibitors from a selected 300 compounds in the ZINC database.

## Methodology:

### Protein and ligand Preparation:

RCSB-PDB was utilized to retrieve the 3D crystal structure of the kinase domain of human HER2 (PDB ID: 3PP0), which was resolved at 2.25 Å [17]. All the ligands were obtained by the ZINC database [18] in the SDF format. The protein and the ligands were prepared using the wizard preparation tool for the docking study.

### Docking Analysis:

For the screening of natural compounds, Autodock Vina was used [19] against the receptor HER2. This Vina program self calculates the grid map and clustering of results displayed for users transparently. In the Vina, a variety of stochastic global optimization methods were discovered, comprising genetic algorithms, particle swarms optimization, and simulated annealing [20]. The active site cavity was selected. Post-docking, energy minimization, and H-bond optimization were carried out. The top 11 best-scored compounds were selected and docked two times. The top 2 best-scored compounds were selected for deep analysis.

### Virtual Screening:

This ZINC database is a free database for virtual screening and it was considered for the retrieval of natural compounds from respective database websites (https://docking.org/). 300 natural compounds were obtained and screened by docking. We can search the compounds in ZINC by numerous search criteria viz. molecular property constraint, ZINC codes, vendor-based and molecular substructure [18].

### SwissADME Analysis:

SwissADME was used for the calculation of ADME, drug-likeness, and pharmacokinetics properties. The graphical output in term of BOILED-Egg was used to represents the passive diffusion through HIA and BBB by position in a WLOGP-versus-TPSA physicochemical space [21].

## Results and Discussion:

This study was performed with the aim of identification of new lead for the inhibition of crystal structure of the kinase domain of HER2, which has expected a potential target for the cure of cancer ailment. Various novel compounds have been designed and developed by employing structure-based computation [22-27]. For the accomplishment of this study, we performed a virtual screening of natural compounds (n: 300) from the ZINC database [18]. After the preparation of all the compounds, a molecular docking study was performed to check the potency of all the selected compounds against HER2 with the reference of the control compound (Lapatinib). Following this process, the top eleven compounds were selected out, which have more free energy of binding (BE) than the control. The list of top selected compounds and their BE were shown in Table 1 (see PDF). Herein, we described the top 2 compounds (ZINC000014780728 and ZINC000014762512) in detail. The BE for the complex 'HER2-ZINC000014780728' and 'HER2-ZINC000014762512' was found to be -11.0 and -10.8 kcal/mol, respectively. The BE for these selected complexes were quite higher than control 'HER2-Lapatinib', which was found to be -10.2 kcal/mol.

**Figure 1:**
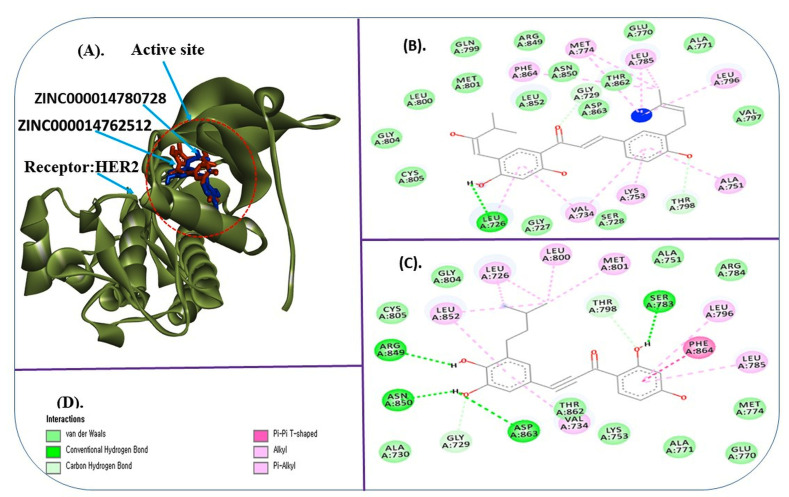
Interacting complex of selected target HER2 with ligands. A) Complex structure of HER2 with ZINC000014780728 (blue) and ZINC000014762512 (red), B) Interacting residues of HER2 active site with ZINC000014780728, C) Interacting residues of HER2 active site with ZINC000014762512. D) Color code represents the different interaction types in figure B&C.

The docking result shows that Leu726, Gly727, Ser728, Gly729, Val734, Ala751, Lys753, Glu770, Ala771, Met774, Leu785, Leu796, Val797, Thr798, Gln799, Leu800, Met801, Gly804, Cys805, Arg849, Asn850, Leu852, Thr862, Asp863, and Phe864 of HER2 catalytic site are the important interacting amino acid (AA) residues with ZINC000014780728 and ZINC000014762512. It has been reported that lapatinib interacts with HER2 through Leu726, Ala751, Glu770, Leu796, and Met801, and in the same study, ZINC15122021 was also reported to interact with Ser728 and Asp863 [16]. Consistent with this study, our selected hit compounds were also found to interact with HER2 with the same AA residues. The complex structure of selected hit compounds against HER2 has shown in Figure 1.

ZINC000014780728 was found to interact through one H-bond with SER726 of HER2, while ZINC000014762512 showing 3 H-bonds with ARG849, ASN850, and Asp863 of HER2. Beside H-bonds, Van der Waals, Pi-Pi, alkyl, and Pi-alkyl bindings were seen to exhibit an important role in the placing of compounds to the active site of HER2 (Figure 1). H-bonds, Van der Waals, Pi-Pi, alkyl, and Pi-alkyl interactions have been reported to stabilize the hit compounds in the HER2 bonding pocket [28]. After that, SwissADME analysis was performed for the top 11 selected compounds along with control. In this, we checked the physicochemical, drug-likeness, and pharmacokinetic properties of the compounds. In this regard, we found that most of the compounds were under the defined parameters of molecular weight (<500Da), hydrogen bond acceptor (≤10), and donor (≤5) [29]. The top two compounds were following the different rules to be drug-likeness, as these are Lipinski, Ghose [30], Veber [31], and Egan [32]. The pharmacokinetics property was showing that the compounds have high gastrointestinal absorption capacity. All these values were shown in (Table 2 - see PDF).

**Figure 2:**
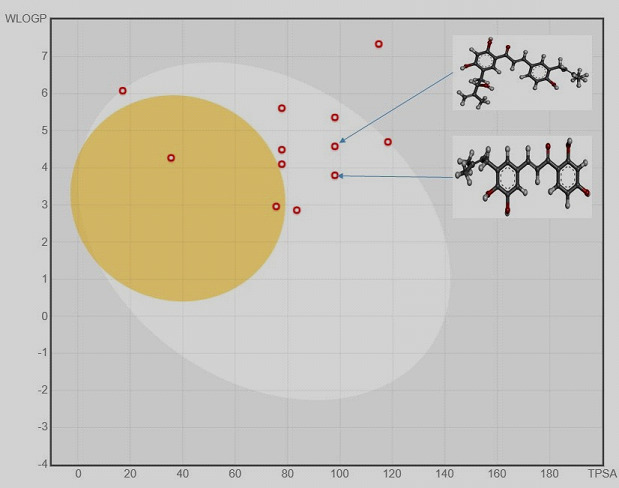
Boiled-egg model of top 11 screened compounds obtained by SwissADME software.

The Boiled-egg model was used to check the property of compounds to be highly gastrointestinal absorption and BBB permeant. Utilizing this model, the top 11 selected compounds were checked. In this, the top 2 selected compounds like ZINC000014780728 and ZINC000014762512 were showing high absorption as indicated in Figure 2. This plot represents the yellow-colored yolk (physiochemical space for highly BBB) and the white space (physiochemical space for HIA absorption). The outputs are based on two descriptors, WLOGP, and TPSA, which is showing the lipophilicity and apparent polarity of the compound [33]. In the docking study, the lesser (high negative) BE reflects the stability of ligand to the corresponding target protein [34]. Interestingly, in this study, compound ZINC000014780728 and ZINC000014762512 show a high affinity for HER2 relative to the control compound (lapatinib) in terms of the BE, suggesting that these two compounds might have the same or increased therapeutic ability corresponding to the reference compound (lapatinib).

## Conclusion

We document the binding features of two compounds (ZINC000014780728 (-11.0 kcal/mol) and ZINC000014762512 (-10.8 kcal/mol)) with HER2 relative to the reference compound (lapatinib (-10.2 kcal/mol)) for further consideration in the context of cancer.
